# Role of Active Components of Medicinal Food in the Regulation of Angiogenesis

**DOI:** 10.3389/fphar.2020.594050

**Published:** 2021-01-22

**Authors:** Dezhi Pan, Xue Gong, Xiaoqin Wang, Minhui Li

**Affiliations:** ^1^ Department of Pharmacy, Inner Mongolia Medical University, Hohhot, China; ^2^ Department of Pharmacy, Baotou Medical College, Baotou, China; ^3^ Pharmaceutical Laboratory, Inner Mongolia Institute of Traditional Chinese Medicine, Hohhot, China; ^4^ Inner Mongolia Key Laboratory of Characteristic Geoherbs Resources Protection and Utilization, Baotou Medical College, Baotou, China

**Keywords:** angiogenesis, food active components, homology of medicine and food, nutritional food, medicinal food

## Abstract

Angiogenesis refers to the formation of new blood vessels from the endothelial cells of existing arteries, veins, and capillaries. Angiogenesis is involved in a variety of physiological and pathological processes, such as the formation of malignant and development of atherosclerosis and other diseases. In recent years, many studies have shown that the active components of food have a certain regulatory effect on angiogenesis and negligible clinical limitations. With the increasing attention being paid to medicine and food homology, exploring the effect of active food components on angiogenesis is of great significance. In this review, we discuss the source, composition, pharmacological activity, and mechanism of action of certain active components of medicinal foods in detail. These could help prevent angiogenesis-related complications or provide a basis for healthier dietary habits. This review can provide a theoretical basis for the research and development of highly efficient anti-angiogenic drugs with low toxicity.

## Introduction

**FIGURE 1:**
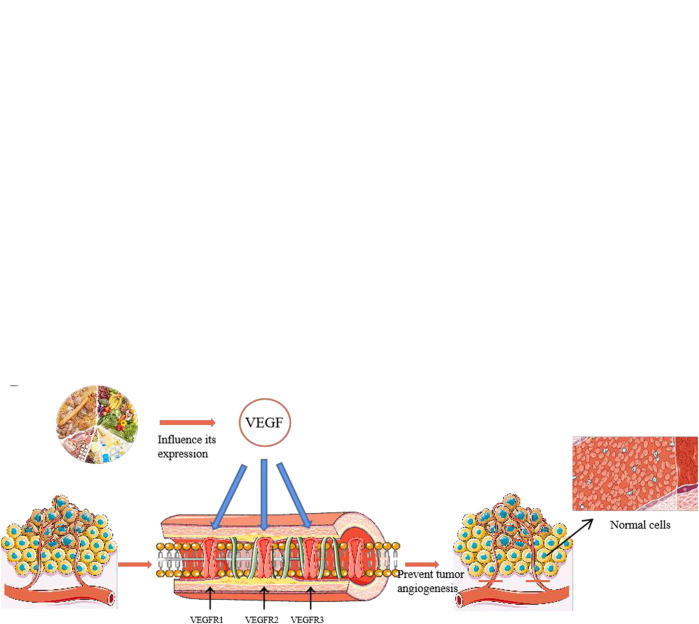
Structure of some flavonoids regulating angiogenesis in medicinal food.

Angiogenesis is a sign of several physiological and pathological states and is the basis of many diseases, such as malignant tumors, cancers, atherosclerosis, and cerebrovascular diseases (Yan and Zhao, 2020). The mechanism underlying angiogenesis is complex and involves several factors, such as transforming growth factor-*β*, platelet-derived endothelial growth factor, cyclooxygenase-2 (COX-2), hypoxia-inducible factor-1, vascular endothelial growth factor (VEGF), and VEGF receptor (VEGFR). Among them, the VEGF, which is a homodimeric glycoprotein encoded by a single gene that can the movement, proliferation, and division of vascular endothelial cells, as well as increase microvascular permeability, is the main angiogenic factor (Yan and Zhao, 2020). The active ingredients in food regulate angiogenesis mainly by affecting VEGF/VEGFR signal transduction (Figure 1A), and can also play an anti-tumor angiogenesis effect by affecting VEGF/VEGFR (Figure 1B).

In the past 20 years, research has shown that tumour angiogenesis plays an important role in tumour growth and that anti-angiogenesis is fundamental in inhibiting tumour growth, invasion, and metastasis (Qin et al., 2017). Therefore, anti-angiogenesis is a good starting point to treat tumors. With the discovery of angiogenesis inhibitors and their accompanying clinical limitations, modern molecular medicine has developed a new field of study in which the anti-angiogenic effects and anti-tumour effects of traditional Chinese medicine are examined and developed (Bagchi et al., 2004). At the same time, it has also been found that drug and food homologous food promote angiogenesis, such as cerebral functional ischemia, through endogenous repair and regeneration is not enough to help the brain recover from cerebral ischemia or brain injury caused by cerebral ischemia, promoting angiogenesis can better solve this problem, and food tonic can also treat cardiovascular and cerebrovascular diseases. Therefore, the medicine-food homology food promote angiogenesis in the treatment of cardiovascular and cerebrovascular diseases and tumor diseases has broad prospects (Yang et al., 2016; Vaeyens et al., 2020). And with the passage of time, people have found the active ingredients in food that can help regulate angiogenesis; and nutritional management of diseases may be easier, safer and more acceptable to patients. Therefore, the research on the role of nutrition in the regulation of angiogenesis has become the main research focus of modern molecular medicine.

Modern clinical trials have shown that the existing angiogenic regulators have certain clinical limitations, such as short therapeutic effects and adverse reactions, which have led to a gradual decrease in the use of anti-angiogenic drugs (Wang et al., 2017; Nikhil et al., 2020). The medical community is committed to finding a better and healthier way to treat angiogenic diseases, therefore, their research direction has gradually changed and now encompasses studies on traditional Chinese medicine and the medicinal properties of food. There are long-standing historical records on the use of food as medicine. The “medicine and food homology” concept is mentioned in the Yellow Emperor’s Classic of Internal Medicine and the Suwen: “fasting food is food, and medicine replaces medicine,” which embodies the theory of medicine and food homology (Shan et al., 2015; Gong et al., 2019; Adelman, 2018). Food can also be used as medicine; when researchers started believing that medicinal foods exert a regulatory effect on angiogenesis, they studied them intensively.

The aim of this study was to provide a comprehensive review of the regulation of angiogenesis by medicinal foods and their active components. To this end, we collated, analysed, and summarised the relevant recent research. This review can be used as a theoretical basis for future research on the regulation of angiogenesis.

## Medicinal Components of Food With Angiogenic Regulation

In this section, we analysed six chemical components of medicinal foods that possess angiogenic activity—flavonoids, terpenes, alkaloids, polyphenols, polysaccharides, and saponins.

### Flavonoids

Flavonoids are natural compounds and secondary plant metabolites with a 2-phenylchromone structure (Li et al., 2016). They have a wide range of pharmacological properties (anti-inflammatory, antibacterial, and anti-tumor), while they are also beneficial for the cardiovascular and central nervous systems. Some studies have shown that flavonoids can regulate angiogenesis, including anti-tumour angiogenesis, as well as promote it. The anti-tumor angiogenesis mechanism entails the down-regulation of matrix metalloproteinases (MMP) (endogenous proteolytic enzyme), VEGF, COX-2, and angiotensin-2 (Huang and Wang, 2019). Figure 1B shows the angiogenic regulatory factors and their anti-tumor mechanisms. The flavonoids from some medicinal plants have been found to regulate angiogenesis. For example, Wang et al. (2019) used zebrafish embryo and rabbit corneal neovascularization models to study the inhibitory effect of isoliquiritigenin on angiogenesis. In particular, they studied whether the triple inhibition of COX-2, microsomal prostaglandin E synthase-1, and cytochrome P450 4A would inhibit tumor angiogenesis through the competing endogenous RNA effect. The results confirmed the anti-angiogenic effect of isoliquiritigenin, and determined that the effect was the most effective at EC50 concentration of 5.9 μmol L ^−1^ (50% concentration for maximum effect) (Wang et al., 2019a).

In another study, the anti angiogenesis effect and mechanism of Puerarin glycosides (PGs) were detected by scratch test, migration test, lumen formation test and cell cycle arrest test. Human umbilical vein endothelial cells (HUVECs) were treated with different concentrations of PGs (100 μg ml^−1^, 200 μg ml^−1^ and 400 μg ml^−1^), and MDA-MB-231 cells were treated with PGs at the same time. Tubulin is an important part of cytoskeleton, and its disordered polymerization hinders the progress of cell cycle. The expression of *α* - tubulin mRNA was detected by RT-PCR-western blot with *α* - tubulin, *β* - actin, cyclin A1 and CDK2 as the main antibodies. The results showed that PGs had anti angiogenic activity and cell cycle blocking ability, and PGs could induce Sphase arrest of MDA-MB-231 cells, inhibit cell proliferation, and the expression level of CDK2 also decreased significantly with the increase of PGs concentration The antiangiogenesis activity of PGA may be related to the ability of cell cycle arrest and the signal pathway of promoting microtubule polymerization. Therefore, PGs have potential antitumor activity (Wu and Jiang, 2010; Li et al., 2018).

The effects of the total flavones of Abelmoschus manihot at different concentrations on angiogenesis were observed using the chick embryo chorioallantoic membrane (CAM) model and gelatin sponges as carriers. The results showed that 5 μg ml^−1^, 10 μg ml^−1^, and 20 μg ml^−1^ of total flavones of A. manihot increased the vascular network and CAM count significantly. This effect was the most obvious at the concentration of 10 μg ml^−1^. Additionally, the total flavonoids of *A. manihot* can protect the heart and brain from ischaemic injury and promote angiogenesis (Pan and Jiang, 2010).

In a study on plant-based diets and plant compounds, the inhibitor effect of 6-methoxy-equol (6-ME) extracted from soybean on tumor angiogenesis was assessed. The HUVECs were either left untreated or treated with 6-ME at different concentrations (1–50 μmol L^−1^) *in vitro*. Quantitative RT-PCR (qRT-PCR) was performed to study the effect of different 6-ME concentrations (0 μmol L^−1^, μmol·L^−1^, and 10 μmol L^−1^) on VEGF expression. The total DNA concentration after isolation was measured. The results showed that 6-ME had the best inhibitory effect at 5 μmol L^−1^. The latest research on the effects of mitogen-activated protein kinase 1/2 and extracellular signal-regulated kinase 1/2 has shown that 6-ME can inhibit tumor angiogenesis by targeting the phosphorylation of mitogen-activated protein kinase 1/2 and its downstream substrate extracellular signal-regulated kinase ½. It thus inhibits the proliferation of ECS induced by VEGF and fibroblast growth factor (FGF) 2 (Sofia et al., 2012).

These studies indicate that the flavonoids in medicinal plants, which play an important role in the regulation of angiogenesis, and the vascular regulation mechanism of flavonoids is mainly affecting the formation of blood vessels in HUVECs and. Some flavonoids have natural hormone like activities. Moreover, compared with other anti-angiogenic drugs, flavonoids are more beneficial to the human body and have no harmful effects. The flavonoids that have an angiogenic regulation ability, as well as their sources, are summarised in Table 1, and the chemical structure of some flavonoids is shown in Figure 1.

**TABLE 1:** Flavonoids with angiogenic regulation.

Source	Bioactive ingredient	Efficacy	Dose	Experimental model	Mechanism	Ref
*Pueraria edulis* Pampan	Puerarin glycoside	Antitumour	100 μg ml^−1^, 10 g kg^−1^	Cell migration and lumen formation	Inhibition of lumen formation of HUVECS	Li et al., 2018, Du et al., 1994
*Glycyrrhiza uralensis* Fisch	Isoliquiritigenin	Antitumour	10 mg kg^−1^	Chick embryo chorioallantoic membrane model	1.Elimination of reactive oxygen (ROS) production and induction of apoptosis in human microvascular endothelial cell (HMEC-1)	Wang et al., 2015c
					2.Inhibition of COX-2, mPGES-1 and CYP4A by isoliquiritigenin blocks the angiogenic Akt signaling in glioma through ceRNA effect of miR-194–5p and lncRNA NEAT1	Wang et al., 2019a
Various fruits and vegetables	Quercetin	Inhibition of retinal and choroidal angiogenesis	—	Cell migration and lumen formation	It inhibits autophagy	Li et al., 2019b
*Epimedium brevicornu* Maxim	Icaritin	Inhibition HUVECs angiogenesis	10^−6^ mol ml^−1^	Cell migration and lumen formation	Decrease the VEGF activity and inhibit the synthesis of vascular endothelial cells, increase the PEDF activity and promote its synthesis	Lv et al., 2014
*Centella asiatica* (L.) Urban	Hispidulin	Antitumour	—	Cell migration and lumen formation	Inhibition of angiogenesis by inhibition of VEGFR2 mediated PI3K/aktmtor signaling pathway	He, 2011
A variety of plants	Apigenin	Antitumour	—	Cell migration and lumen formation	Down regulate the expression of hypoxia inducible factor-1(HIF-1a) by inhibiting the binding of HSP90 and HIF-1a in ovarian cancer	Fang et al., 2007
*Glycine max* (L.) Merr	Soybean isoflavone	1.Antitumour 2.Inhibition of angiogenesis in transplanted hepatoma	—	Animal model of transplanted tumor	1.Inhibition of angiogenesis 2.Down regulation of VEGF and transforming growth factor-*β* (TGF - *β*) 1 protein expression in transplanted tumor	He et al., 2003 Zheng et al., 2018
*Glycine max* (L.) Merr	Genistein	Treatment of breast cancer	—	—	By down regulating the expression of VEGF, it can inhibit the angiogenesis of breast cancer with high expression of HER-2/neu gene. The inhibition of angiogenesis is related to the inhibition of the signal transduction pathway of tylosin kinase membrane receptor tyrosine protein kinase (TPK)	Yu et al., 2003
*Abelmoschus manihot* (L.) Medicus	Total flavone of Abelmoschl Manihot Lmedi (TFA)	Protect the ischemic injury of heart and brain and promote angiogenesis	10 g ml^−1^	Animal model of transplanted tumor	Promoting angiogenesis	Pan and Jiang, 2010
*Morus alba* L	Cortex Mori flavone extracts	Treatment of nonalcoholic fatty liver (NAFLD)	1.0 g kg ^−1^	Model of type 2 diabetes mellitus with nonalcoholic fatty liver disease	Inhibition of VEGF and platelet derived growth factor (PDGF) mRNA expression in liver	Qin et al., 2017
*Myrica rubra* Siebold et Zuccarini	Dihydromyricetin	Antitumour	30 mg kg^−1^	Animal model of transplanted tumor	By inhibiting ERK/VEGFA/VEGFR2 signaling pathway, it can inhibit angiogenesis	Wen et al., 2020
	Myricetin	Antitumour			And Akt/p70s6khif-1 α/VEGF protein expression	Huang 2015
	Koryo curcumin	Antitumour				
	kaempferol	Antitumour				
*Glycine max* (L.) Merr	Daidzein	Antitumour	—	—	Inhibition of angiogenesis	Kang et al., 2015
*Alpinia officinarum* Hance	Koryo curcumin	Antitumour and antithrombotic	20 mg·(kg·d)^−1^ 40 mg·(kg·d)^−1^	Implanted tumor model of hepatocellular carcinoma in nude mice	Inhibition of angiogenesis	Hua et al., 2017
*Myrica rubra* Siebold et Zuccarini	Anthocyanidin Genistein	Antitumour Antitumour	—	—	Inhibition of basic MCP1 and NF-kB transcription significantly reduces angiogenesis *in vivo*	Bagchi et al., 2003; Feng et al., 2014a Fotsis et al., 1995
*Glycine max* (L.) Merr	Genistein	Antitumour	—	—	Affect the expression of VEGF and inhibit angiogenesis	Xing et al., 2014 Yang et al., 2009 Huang et al., 2007
	6-methoxyequol (6-ME)	Antitumour	5 μmol L ^−1^	Cell migration assay	Inhibition of VEGF and FGF2 induced ECs proliferation	Sofia et al., 2012
*Poncirus trifoliata* (L.) Raf	Poncirin	Antitumour and inhibit angiogenesis	12.5 μmol L ^−1^ 25 μmol L ^−1^	Chick embryo chorioallantoic membrane model	Inhibition of vascular endothelial cell proliferation and decrease of VEGF expression in tumour cells	Li et al., 2016

**TABLE 2:** Terpenoids with angiogenesis regulation.

Source	Bioactive ingredient	Efficacy	Dose	Experimental model	Mechanism	Ref
*Curcuma phaeocaulis* Valeton	Curcumol	Promote angiogenesis	20 mg ml^−1^ 1 mg ml^−1^ 50 mg ml^−1^	Biological model experiment of zebrafish embryo	Promote angiogenesis and repair of tissue damage through VEGF pathway	Tian et al., 2012
*Bletilla striata* (Thunb. ex Murray) Rchb. F	Cyclotunane triterpenoids	Antitumour	50 μg ml^−1^, 100 μg ml^−1^	Angiogenesis model of rat arterial ring	Antagonizing the proliferation and inducing apoptosis of HUVECs stimulated by VEGF and bFGF	Liu et al., 2008b Liu et al., 2009 Liu et al., 2015b Liu et al., 2016
Widespread in plants and herbs	Oleanolic acid	Antitumour	—	—	Inhibit the expression of VEGF in liver cancer, reduce the level of VEGF, so as to play the role of inhibiting tumor growth	Harper and Moses Marsha 2006
*Cornus officinalis* Sieb. et Zucc	Ursolic acid	Antitumour	10 mg L^−1^ 15 mg L^−1^	Biological model test of zebrafish	Blocking VEGFR2 expression	Cheng et al., 2010 Liu et al., 2012
*Vigna unguiculata* (L.) Walp	3*-O-*Acetyloleanolic acid	Antitumour	—	—	Induction of HUVECs apoptosis and inhibition of angiogenesis	Cui et al., 2013
*Panax notoginseng* (Burkill) F. H. Chen ex C. Chow & W. G. Huang	Notoginseng triterpenes	Anti-angiogenesis	0.4 mg L^−1^ 0.8 mg L^−1^	EA-hy926 cells transplanted *in vitro*	Anti-angiogenesis	Shi et al., 2013
*Salvia miltiorrhiza* C. H. Wrigh	Dihydrotanshinone I	Anti tumour and ischemic diseases	—	—	Inhibit the proliferation, migration, invasion and lumen formation of vascular endothelial cells, thereby inhibiting angiogenesis	Wang et al., 2015a Wei et al., 2010
	Tanshinone I	Antitumour and ischemic diseases	—	—	Inhibition of VEGF expression	Wang et al., 2015b
	Tanshinone IIA	Antitumour and ischemic diseases	1 μmol L^−1^ 40 mmol L^−1^	Chick embryo chorioallantoic membrane model	1.Promote the expression of HIF-1*α* mRNA to up regulate the expression of VEGF 2.Tanshinone VI can up regulate cell adhesion molecules, thus inhibiting metastasis or ngiogenesis 3.Inhibition of matrix invasion and modification of MMP-2/TIMP-2 secretion in vascular endothelial cells	Chen, 2007 Tsai et al., 2011 Wang et al., 2015a
	Cryptotanshinone	Antitumour and ischemic diseases	—	—	Inhibition of basic fibroblast growth factor (bFGF) -induced angiogenesis	Wang et al., 2015a

### Terpenoids

**FIGURE 2:**
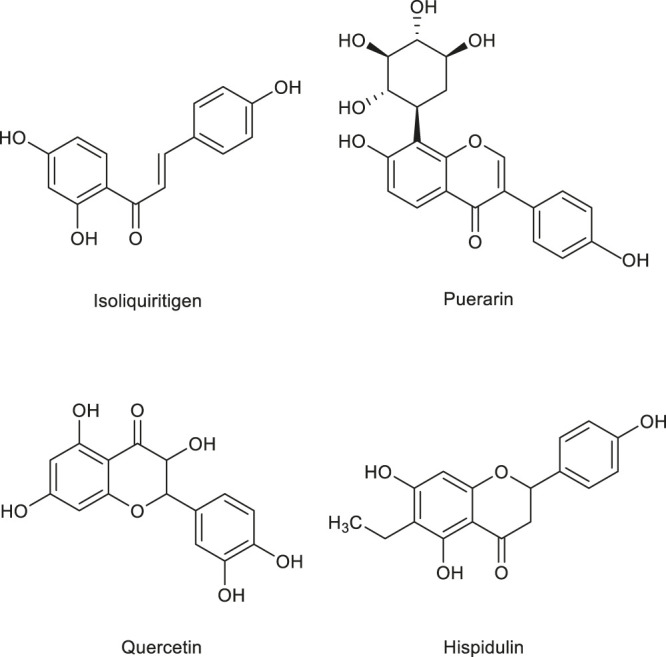
Structure of some terpenoids regulating angiogenesis in medicinal food.

Terpenoids are natural hydrocarbons, which are abundant in nature and can be linked by isoprene or isopentane units in various ways. All kinds of terpenoids have been proved to be effective chemical raw materials and have significant disease prevention and treatment effects. Especially, they show good antitumor activities. They have potential to be used as lead compounds to develop efficient and safe new antitumor drugs. In addition, studies have shown that some terpenoids can regulate angiogenesis and have a good effect on the treatment of cardiovascular diseases. Therefore, further study on the angiogenesis regulation of terpenoids not only contributes to the development of new anti-tumor drugs, but also has great advantages in the treatment of angiogenesis related diseases. Therefore, terpenoids have great application potential and broad development prospects due to their special structure and function as well as extensive medical uses (Zhang et al., 2018). For this reason, some terpenoids with angiogenic effect (Table 3) are sorted out and the structural diagram of some terpenoids is shown (Figure 2).

**TABLE 3:** Saponins with angiogenesis regulation.

Source	Bioactive ingredient	Efficacy	Dose	Experimental model	Mechanism	Ref
*Panax ginseng* C. A. Meyer	Ginsenoside Rg1	Treatment of acute myocardial infarction	1 mg kg^−1^, 5 mg kg^−1^	Animal model of transplanted tumor	Increase VEGF expression and stimulate angiogenesis in myocardial infarction area	Jin and Liu 2007
*Talinum paniculatum* (Jacq.) Gaertn	Ginsenoside Rg3	1.Antitumour 2.Inhibition of angiogenesis mimicry and migration of CNE-2 cells *in vitro*	5 μg ml^−1^	Animal model of transplanted tumor	1. Inhibit angiogenesis and down regulate the expression of MMP-2 and MMP-9 2. Inhibition of COX-2, HIF-1 VEGF and fascinl protein expression in CNE-2 cells	Xin et al., 2010 He et al., 2012b Kong et al., 2015 Guo et al., 2014 Guo and Lin, 2017 Geng et al., 2016
*Panax ginseng* C. A. Meyer	Ginsenoside Rh2	Antitumour	1.0 mg · kg^−1^ 3.0 mg · kg^−1^	Animal model of transplanted tumor	Decrease VEGF expression	Wu, 2014 Cui and Qu, 2011, Si et al., 2020
*Panax ginseng* C. A. Meyer	Ginsenoside Rb1	Improving cardiac function	—	—	Activation of HIF-1*α* to promote angiogenesis	Kimura et al., 2006
*Platycodon grandiflorus* (Jacq.) A. DC.	Saponins (SCPG)	Antitumour	100 μg ml^−1^	Chick embryo chorioallantoic membrane model	Inhibit the expression of VEGF, thus affecting the balance between tumour growth factor and inhibitory factor	Yuan et al., 2014
*Panax notoginseng* (Burkill) F. H. Chen ex C. Chow & W. G. Huang	Panax notoginseng saponins	1.Improving myocardial ischemia 2. Antitumour 3. Improving postmenopausal osteoporosis	—	—	1. Stimulates HIF reverse transcription activity, cardiomyocyte VEGF、bFGF protein expression 2. Inhibition of angiogenesis 3. Inhibition of angiogenesis	Wang et al., 2014a Wang and Zhang, 2013
*Arctium lappa* L	Arctigeni	Pathological angiogenic diseases	10.0 g L^−1^	Chick embryo chorioallantoic membrane model	Inhibition of angiogenesis	Liu et al., 2014a
*Rhodiola rosea* L	Salidroside	Antitumour	—	—	Inhibition of angiogenesis	Zhu et al., 2012
*Panax notoginseng* (Burkill) F. H. Chen ex C. Chow & W. G. Huang	Notoginsenoside R1	Angiocardiopathy	—	—	Promoting angiogenesis	Yang et al., 2016

The zebrafish biological model has been used to observe the effects of curcumol on the vascular growth of embryonic bodies, the vascular regeneration of adult fish after cutting their tails, and the tissue regeneration of larvae after cutting their tails. VEGF and VEGFR2 gene expressions were detected by relative quantitative fluorescence PCR. For this, zebrafish embryos were treated with different curcumol concentrations (1 mg L^−1^, 5 mg L^−1^, 10 mg L^−1^, 20 mg L^−1^, and 50 mg L^−1^). The results showed that 20 mg L^−1^ promoted the vascular endothelial growth of zebrafish embryo internodes, whereas 50 mg L^−1^ promoted VEGFR2 growth in zebrafish embryos. Moreover, 1 mg L^−1^ curcumol had the strongest effect on tissue regeneration. Therefore, curcumol could promote angiogenesis by promoting the expression of VEGF and VEGFR2 (Tian et al., 2012).

Additionally, some studies have examined, by fluorescence microscopy, the effects of different doses of ursolic acid (5 mg L^−1^, 10 mg L^−1^, and 20 mg L^−1^) on the formation of intersegmental vessels on the backs of AB and transgenic fluorescent zebrafish (VEGFR2:GFP) that were used as experimental animals. The results showed that at ursolic acid doses of 5 mg L^−1^ and 10 mg L^−1^, intersegmental vessel proliferation induced by transplanted cancer was inhibited significantly, whereas the inhibition of angiogenesis and VEGFR2 was also possible. It has been suggested that ursolic acid significantly inhibits the physiological characteristics of zebrafish and the angiogenesis caused by transplanted tumors, which is related to the inhibition of VEGFR2 (Cheng et al., 2010).

Furthermore, the anti-angiogenic effect of different tanshinone IIA (Tan ⅡA) doses (1 μmol L^−1^, 5 μmol L^−1^, and 10 μmol L^−1^) on chicken embryos and human umbilical vein endothelial cells (HUVECs) was studied using the CAM model. The mRNA expression of MMP-2, MMP-3, MMP-9, and MMP-14, tissue inhibitor of metalloproteinase, and reversion-inducing cysteine-rich protein was detected by RT-PCR. The results showed that Tan IIA inhibits angiogenesis on chicken embryos and HUVECs, and increasing Tan IIA concentration decreases the inhibitory effect. The results of western blotting and ELISA further confirmed that after Tan ⅡA treatment, MMP-2 decreased in a dose-dependent manner, whereas the level of tissue inhibitor of metalloproteinase secretion increased. These results suggest that Tan ⅡA has anti-angiogenic effects both *in vivo* and *in vitro*. Additionally, its mechanism of action is related to its inhibitory effect on MMP-2, whereas it has the opposite regulatory effect on the tissue inhibitor of metalloproteinase secretion, thereby decreasing the MMP-2 activity in vascular endothelial cells (Tsai et al., 2011).

### Saponins

**FIGURE 3:**
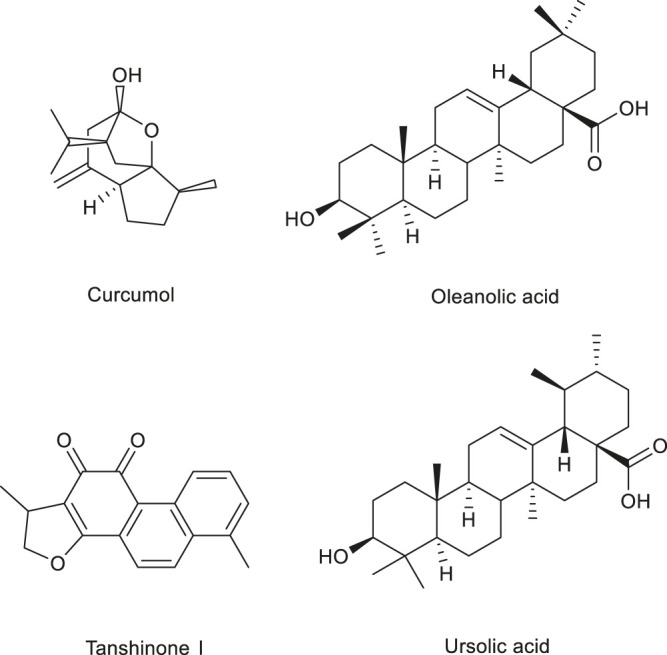
Structure of some saponins regulating angiogenesis in medicinal food.

Saponins are glycosides composed of triterpenoids or spiral sterane compounds and are widely distributed in nature, including in monocotyledons and dicotyledons (Liu and Henkel, 2002). Saponins have important physiological and pharmacological activities. In recent years, scholars have been actively looking for angiogenesis regulators from natural drug sources. As natural active ingredients in food, saponins have been tested in animal models and have been found to be safe and regulate angiogenesis. The saponins that regulate angiogenesis are shown in Table 3, and the structure of few saponins is shown in Figure 3.

Ginsenoside Rg1 and RB1 have been reported to regulate cardiac function and promote angiogenesis, while ginsenoside Rh2 has antitumor and antiangiogenic effects (Jin and Liu, 2007).

Some studies have divided the rats into sham operation group, acute myocardial infarction control group, ginsenoside Rgl low-dose treatment group (1 mg kg^−1^) and high-dose treatment group (5 mg kg^−1^) to study the angiogenesis promoting effect and mechanism of Ginsenoside Rg1. Myocardial enzymes, infarct size and microvessel density were measured at different time points. The expression of VEGF-mRNA was detected by RT-PCR. The results showed that the microvessel density and VEGF-mRNA expression in the sham operation group were lower than those in the operation group at different time points. In addition, myocardial enzyme activity and infarct size in the treatment group were significantly lower than those in the control group, and the degree of angiogenesis in the infarct area continued to increase steadily, with significant difference compared with the control group. In conclusion, severe myocardial ischemia can stimulate the production of a large amount of VEGF, and the difference in each experimental group can show the mechanism of ginsenoside Rgl in promoting angiogenesis, which is related to the increase of VEGF-mRNA expression in myocardial infarction area (Jin and Liu, 2007).

Additionally, the anti-tumor mechanism and effect of ginsenoside Rh2 on Lewis lung carcinoma in mice was studied using a solid tumor model of the carcinoma. The anti-tumor effect of ginsenoside Rh2 and VEGF expression in the tumor were observed by immunohistochemistry. The experimental group was administered different Rh2 doses (0.3 mg kg^−1^, 1.0 mg kg^−1^, and 3.0 mg kg^−1^), and a cyclophosphamide group (20 mg kg^−1^) was established. The results showed that the tumor weight and the microvessel density in the ginsenoside Rh2 group was significantly lower than that in the control group. VEGF protein was mainly expressed in the new capillaries of tumor cells and some tumor cell membranes and plasma that were stained. The results demonstrate that the positive rate of VEGF protein expression decreases with the increase in ginsenoside Rh2 concentration. Therefore, the inhibitory effect of ginsenoside Rh2 on tumor angiogenesis can be induced by inhibiting the expression of VEGF protein, thereby inhibiting the release of VEGF and tumor angiogenesis (Cui and Qu, 2011).

### Polysaccharides

Polysaccharides, which consist of 10 or more single sugar molecules polymerised by a glucosidic bond, have relatively high molecular weight and generally consist of hundreds or even tens of thousands of single sugar molecules. Polysaccharides are important biological macromolecules, which exist widely in animals, plants, and microorganisms. The biological activities of polysaccharides have recently attracted considerable attention in biochemical and medical research because of their immunomodulatory effects (Lu et al., 2000). Polysaccharides have a wide range of biological activities and low toxicity as well as have a good potential for the development of clinical drugs. Therefore, they can be potentially used for the treatment of angiogenic diseases. In this section, we collated studies that have reported the regulation of angiogenesis by polysaccharides (Table 4).

**TABLE 4:** Polysaccharide compounds with angiogenesis regulation.

Source	Bioactive ingredient	Efficacy	Dose	Experimental model	Mechanism	Ref
*Holothuria leucospilota*	Mucoitin sulfate	Anticoagulant, antithrombotic	—	—	Inhibition of bud formation, formation of vascular network and proliferation of endothelial cells	Lu et al., 2004
*Grossulariaceae*	Phellinus ribis polysaccharide	Antitumour	—	—	Inhibition of angiogenesis	Xv et al., 2016
*Coriolus versicolor* (L.exFr.)Quel	Polysaccharide of *Coriolus versicolor*	Treatment of atypical hyperplasia of the breast	3.3 mg kg^−1^	Experimental model of mammary gland atypical hyperplasia induced by 7,12-dimethylbenzoanthracene in SD rats	To inhibit the expression of VEGF and ras in breast hyperplasia and reduce angiogenesis	Wu et al., 2001
*Saccharum officinarum* L	Sugarcane leaves polysaccharide	Reduction of myocardial infarction area	—	—	Facilitate VEGF expression and microvascular formation	He et al., 2016
*Hedysarum polybotrys* Hand. -Mazz	Hedysarum Polybotys saccharide	Treatment of angiogenic disorders	—	—	Promoting angiogenesis	Liu et al., 2008a
*Angelica sinensis* (Oliv.) Diels	Angelica sinensis polysaccharide	—	2 mg ml^−1^	Chick embryo chorioallantoic membrane model	Promoting angiogenesis	Niu et al., 2009
*Aloe vera*	Aloe polysaccharid (AP)	Antitumour	71.74 mg·l00g^−1^	—	Anti-tumour neovascularization	Lu and Cai 2012
*Ganoderma Lucidum* (Leyss. ex Fr.)Karst	Ganoderma lucidum polysaccharide	Antitumour	3.188 mg ml^−1^	T24 tumor bearing nude mice model	Down-regulated expression of VEGF and bFGF	Guo et al., 2014
*Lentinus edodes* (Berk.)sing	Lentinan	Antitumour	500 mg L^−1^	Cell migration experiment	Inhibition of angiogenesis	Zhu, 2017
*Schisandra chinensis* (Turcz.) Baill	Schisandra chinensis polysaccharide	Antitumour and vascular inhibition	—	—	Depress VEGF secretion	Zhao et al., 2019
*Laminaria japonica* Aresch	Laminarin	Antitumour	9.5 μg ml^−1^	Chick embryo chorioallantoic membrane model	Inhibition of angiogenesis	Xv et al., 1999
*Prunella vulgaris* L	*Prunella vulgaris* sulfated polysaccharide	Antitumour	—	—	Blocking the formation of bFGF receptor trinets and inhibiting bFGF secretion, thereby inhibiting vascular endothelial cell growth	Wang et al., 2014
*Sanghuangporus lonicericola *(Parmasto) L. W. Zhou	Mulberry polysaccharide	Antitumour	—	—	Expression of inhibitory VEGE	Zhao 2007, Lv et al., 2014
*Carthamus tinctorius* L	Safflower polysaccharide (SPS)	Antitumour	—	—	To inhibit VEGF expression, reduce MVD, inhibit angiogenesis of tumour tissue	Liang 2012
*Lycium chinense* Miller	Lycium barbarum polysaccharide (LBP)	Prevention of retinal microvascular disease	—	—	Inhibit VEGF expression, thereby inhibiting angiogenesis	Tian et al., 2013
*Phaeophyta*	Fucosan	Anticoagulant, Antithrombotic	—	—	Depress VEGF expression and angiogenesis	Satoru et al., 2003, Xin et al., 2017

In one study, the effect of five different lentinan concentrations (31.25 mg L^−1^, 62.5 mg L^−1^, 125 mg L^−1^, 250 mg L^−1^, and 500 mg L^−1^) and thalidomide (200 mg L^−1^) as the positive control, on HUVEC proliferation was examined using the MTT method. Additionally, the transwell cell migration test, FN adhesion test, and tuber formation assay were used to detect the effect of lentinan on cell migration, cell adhesion, and angiogenesis *in vitro*, respectively. The results showed that lentinan inhibits cell migration, cell adhesion, and angiogenesis in a dose-dependent manner, with the most significant inhibitory effect produced by the 500 mg L^−1^ concentration. The anti-tumour effect of lentinan is related to its inhibitory effect on HUVEC proliferation, migration, adhesion, and angiogenesis *in vitro* (Zhu, 2017).

There are studies on the inhibition of *G. lucidum* polysaccharide on tumor angiogenesis and its mechanism. The effect of *G. lucidum* polysaccharide combined with cisplatin on the proliferation of human bladder cancer cell line T24 *in vitro* was determined by MTS method, and the T24 tumor bearing nude mice model was established to observe the combined treatment effect of *G. lucidum* polysaccharide and cisplatin. The microvessel density (MVD) and the expression levels of vascular endothelial growth factor (VEGF) and basic fibroblast growth factor (bFGF) were detected by immunohistochemistry Real time PCR and Western blotting were used to detect the expression of VEGF and bFGF. The results showed that *G. lucidum* polysaccharide could effectively inhibit the proliferation of T24 cells *in vitro*, and had synergistic effect with cisplatin. Moreover, *G. lucidum* polysaccharide (3.188 mg L^−1^) could significantly enhance the inhibitory effect of cisplatin on tumor growth of nude mice, inhibit the angiogenesis of tumor tissue and the expression of VEGF and bFGF. Therefore, *G. lucidum* polysaccharide can inhibit the growth and angiogenesis of T24 tumor bearing nude mice, and its mechanism may be related to the down-regulation of VEGF and bFGF expression (Guo et al., 2014).

### Alkaloids

Alkaloids are a class of nitrogen-containing organic compounds found in nature and derived mainly from plants. Most alkaloids have complex ring structures on which the nitrogen atoms are bound. Additionally, most alkaloids are basic, have significant biological activities, and are important pharmaceutical bioactive components in medicinal food (Jin et al., 2004). It has been confirmed that plant alkaloids have a wide range of anti-tumour and anti-angiogenesis effects and can be obtained from a wide range of sources; therefore, they can be potentially used to treat various clinical conditions (Feng and Tang, 2014).

In one study, the authors isolated, purified and identified the active components from the fruit of Evodia rutaecarpa. Through the zebrafish experimental model method, nine of them were detected to have angiogenesis inhibitory effect (Yin et al., 2016).

**FIGURE 4:**
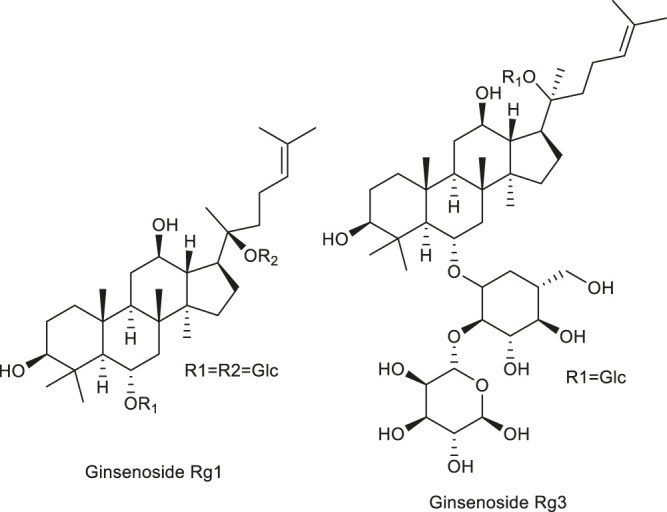
Structure of some alkaloids regulating angiogenesis in medicinal food.

The antitumor effect of 5,2,4′- trihydroxy-6,7,5' - trimethoxyflavone (TTF1) was studied in this paper. The mechanism of TTF1 action included inhibition of tumor angiogenesis and induction of tumor cell apoptosis. In order to further study the molecular mechanism of its inhibitory effect on tumor angiogenesis, cam method was used to determine the angiogenesis inhibitory effect of TTF1. In order to detect whether the angiogenesis inhibitory effect of TTF1 is related to the expression of VEGF, KDR, bFGF, COX-2 and HIF-1α, the protein levels of these factors in hepg-2-induced mouse tumor were measured The expression of VEGF, KDR, bFGF, COX-2 and HIF-1α was decreased by TTF1 at different concentrations (5 μmol kg^−1^, 10 μmol kg^−1^ and 20 μmol kg^−1^), respectively White matter level to achieve anti angiogenesis effect (Liu et al., 2011a). Table 5 shows the recent research progress on the anti-angiogenic mechanisms of food alkaloids. Figure 4 lists the structural formulas of alkaloids in two common medicinal foods.

**TABLE 5:** Alkaloids with angiogenic regulatory action.

Source	Bioactive ingredient	Efficacy	Dose	Experimental model	Mechanism	Ref
*Evodia rutaecarpa* (Juss.) Benth.	Rutaecarpine	Antitumour	—	—	Inhibition of angiogenesis	Yin et al., 2016
	Evodiamine	Antitumour	—	—	Inhibition of angiogenesis	
	Goshuyuamide-Ⅰ	Antitumour	—	—	Inhibition of angiogenesis	
	*N*-formyldihydrorutaecarpine	Antitumour	—	—	Inhibition of angiogenesis	
	1-Methyl-2-undecyl-4 (1H) -quinolone	Antitumour	—	—	Inhibition of angiogenesis	
	Dihydroevocarpine	Antitumour	—	—	Inhibition of angiogenesis	
	*N-*methylanthranylamide	Antitumour	—	—	Inhibition of angiogenesis	
	Limonoids calodendrolide	Antitumour	—	—	Inhibition of angiogenesis	
*Coptis chinensis* Franch	Berberine	Antitumour	5 μmol L^−1^	Chick embryo chorioallantoic membrane model	Targeting binding and inhibiting VEGFR2 activity, blocking its mediated activation of AKt/mTOR/P70S6K signaling pathways, and then acting as an antiangiogenic agent	Li et al., 2019c; Wang et al., 2014
*Crataegus pinnatifida* Bge	5,2,4′-trihydroxy-6,7,5′-trimethoxyflavone (TTF1)	Antitumour	5 μmol kg^−1^ 10 μmol kg^−1^ 20 μmol kg^−1^	Chick embryo chorioallantoic membrane model	Down regulation VEGF, KDR, bFGF, HIF-1α and COX-2	Liu et al., 2011a
*Capsicum annuum* L	Capsaicin	Antitumour	—	—	Inhibition of angiogenesis	Liu et al., 2014
*Corydalis yanhusuo* W. T. Wang	Alkaloids	Anti-angiogenesis	—	—	Targeted inhibition of vascular endothelial growth factor receptor signaling	Wan et al., 2020

### Polyphenols

Polyphenols (tannins) are important natural products that are found widely in plants. Polyphenols have a complex structure and active chemical properties and their structure comprises many homologous compounds; therefore, research on these compounds has progressed relatively slowly (Song et al., 2000). However, in the past 30 years with the development of polyphenol chemistry, the chemical structure and properties of polyphenols have been revealed in detail. Plant polyphenols are recognised as safe anti-tumour agents that act via several mechanisms, including affecting tumour angiogenesis. Moreover, polyphenols have antioxidant, antimutagenic, anticancer, and anti-inflammatory properties, thus ensuring the stability of the human genome and preventing occurrence of diseases. Therefore, polyphenols present in some foods can be potentially used as therapeutic agents (Anupama et al., 2002; Wang et al., 2005).

The effect of emodin on human liver cancer has been studied by examining the angiogenesis of HepG2 cells. To this end, the effects of HIF1A and VEGF on HepG2 cells, as well as the anti-hepatoma effects of rhein, were examined using an *in vivo* chicken CAM model. The experiment included randomly divided groups: a negative control group (normal saline), emodin low-dose (10 μmol L^−1^) and high-dose groups (20 μmol L^−1^), and a positive control group (0.15 mg L^−1^; dexamethasone). The inhibitory effect of emodin on CAM angiogenesis was observed using *in vitro* culture methods. For this, HepG2 cells were treated with CoCl2 to simulate chemical hypoxia. Hypoxia untreated (negative control group [normal saline]), hypoxia emodin low-dose (10 μmol L^−1^), hypoxia emodin high-dose (20 μmol L^−1^), and hypoxia positive control groups (10 μmol L^−1^; 5-fluorouracil) were established. After 24 hours of treatment, the expression of HIF1A and VEGF was detected by immunocytochemistry, and the expression of HIF1A and VEGF mRNA was detected by qPCR. The CAM results showed that the number of neovascularization in the experimental group was significantly reduced, especially in the low-dose group (10 μmol L^−1^), the HIF1A and VEGF positive cells in the low-dose, high-dose and positive control groups were significantly reduced; the HIF1A and VEGF positive cells in the low-dose group (10 μmol L^−1^) were significantly reduced, and the levels of HIF1A and VEGF mRNA in the experimental group were lower than those in the negative control group. Therefore, it can be concluded that emodin may inhibit the angiogenesis of HepG2 cells by inhibiting the expression of HIF1A mRNA, thereby also reducing VEGF mRNA level (Gao et al., 2016a).

In another study, the regulation of chlorogenic acid on cox2-mmp signaling pathway in transgenic zebrafish was studied. Therefore, 100 fli1a EGFP transgenic zebrafish embryos were randomly divided into blank group, positive control group and chlorogenic acid high, medium and low dose groups. 20 positive controls in each group were treated with PTK787 5 pg ml^−1^, chlorogenic acid high, medium and low dose groups were treated with chlorogenic acid 200 μg ml^−1^, 100 μg ml^−1^ and 50 μg ml^−1^, respectively. Twenty six hours after treatment, vascular phenotypes were observed and photographed with stereomicroscope (bright field) and stereofluorescence microscope. The expression levels of COX-2 mRNA, MMP-9 mRNA and MMP-2 mRNA in zebrafish embryos were detected by real-time PCR. The results showed that compared with the blank group, the vascular inhibition was obvious in chlorogenic acid group. The levels of COX-2 mRNA, MMP-9 mRNA and MMP-2 mRNA in chlorogenic acid and ptk797 groups were significantly lower than those in the blank control group. The levels of MMP-9 mRNA and MMP-2 mRNA in high-dose chlorogenic acid group were significantly lower than those in medium dose chlorogenic acid group, while those in medium dose chlorogenic acid group were significantly lower than those in low-dose chlorogenic acid group. It is concluded that chlorogenic acid can inhibit the angiogenesis of transgenic zebrafish embryos. The mechanism of action is related to the down regulation of cox-2-mmp signal pathway and the inhibition of MMP-9 and MMP-2 gene expression (Cai et al., 2019).

**FIGURE 5:**
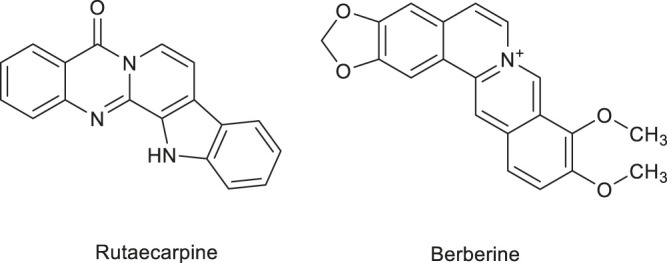
Structure of some polyphenols regulating angiogenesis in medicinal food.

The results of the aforementioned studies demonstrate that the polyphenols found in some medicinal plants have a certain regulatory effect on angiogenesis. Table 6 shows current progress on the regulation of angiogenesis by polyphenols. Figure 5 lists the structural formulas of common polyphenols present in several medicinal foods.

**TABLE 6:** Polyphenols with angiogenesis regulation.

Source	Bioactive ingredient	Efficacy	Dose	Experimental model	Mechanism	Ref
*Curcuma longa* L	Curcumin	Antitumour	—	—	1.Inhibition of vascular endothelial cell proliferation and promotion of apoptosis 2.Expression of angiogenesis Promoter 3.Down-regulation of PI3K/AKT and mitogen-activated protein kinases (MAPK)	Gao et al., 2016b; Li et al., 2020, Shankar et al., 2008, Hishe et al., 2011, Dorai et al., 2001, Zhang 2013, Gururaj et al., 2002, Zheng et al., 2007
*Rheum palmatum* L	Emodin	Antitumour	10 μmol L^−1^	Chick embryo chorioallantoic membrane model	Inhibit the expression of HIF-1αmRNA, thereby reducing the expression of EGF mRNA	Gao et al., 2016
*Curcuma longa* L	Curcumin Ⅲ	Antitumour	—	—	Inhibition of angiogenesis in tumour tissue	Mohan et al., 2000
*Lonicera japonica* Thunb	Chlorogenic acid	Anti-angiogenesis	50 μg ml^−1^	Biological model experiment of zebrafish embryo	Down-regulation of COX2-MMPs signaling pathways, thereby inhibiting MMP-9 and MMP-2 gene expression	Cai et al., 2019, Su et al., 2014
Green tea	Tea polyphenols	Antitumour	11.25 mg kg^−1^	Transplantation tumor model in S10 mice	1.Inhibition of angiogenesis 2.Regulation of MMP-2/TIMP-2 by regulating NF-kB signaling pathways	Lu and Cao, 2001 Zhang et al., 2007, Chen, 2007, Zhang 2007, Huang 2007
	Epigallocatechin-3-gallate	Antitumour	—	—	The inhibition of cell MMP-2 and MMP-9 secretion also inhibits the expression of VEGF、 phosphorylated VEGF receptor (P.VEGFR)-2 protein and the activation of downstream signaling molecules extracellular signal-regulated kinases (extracellularsignal -Regula-tedkinase,ERK) and Akt	He et al., 2012
	Catechin	Antitumour	—	—	Anti-angiogenesis	Liu et al., 2015
*Salvia miltiorrhiza* Bunge	Salvianolic acid B	1.Anti-myocardial ischemia 2.Treatment of neurodegenerative diseases	1.2 mg L^−1^	Cell migration and lumen formation, chick embryo chorioallantoic membrane model	1.Upregulate the expression of VEGF in HUVECS, thus promoting angiogenesis 2.Promote angiogenesis	Xv et al., 2016, Li et al., 2010, Tang et al., 2014
*Rosmarinus officinalis* L	Rosmarinci acid	Antitumour	—	—	Inhibit the expression of related VEGF and release of IL-8, thereby inhibiting angiogenesis	Huang and Zheng, 2006
	Carnosol	Antitumour	—	—	Inhibit endothelial cell migration and inhibit BAEC secretion MMP-2, thereby inhibit angiogenesis	Auxiliadora et al., 2013
	Carnosic acid	Antitumour	—	—	Inhibit endothelial cell migration and inhibit BAEC secretion MMP-2, thereby inhibit angiogenesis	
*Salvia miltiorrhiza* Bunge	Danshensu	Prevention of retinal microvascular disease	200 μmol L^−1^	Biological model experiment of zebrafish embryo	Inhibit VEGF expression, thereby inhibiting angiogenesis	Tian et al., 2013 Cui et al., 2013
*Fragaria × ananassa*	Strawberry phenols	Antitumour	—	—	Inhibit VEGF expression, thereby inhibiting angiogenesis	Wang et al., 2012
*Vitis vinifera*	Procyanidin	Antitumour	223. 24 U·ml^−1^	Biological model experiment of zebrafish embryo	Inhibit VEGF-A and VEGFR2 signaling pathways, thereby inhibiting angiogenesis	Li et al., 2016, Zhang, 2017, Hishe et al., 2011; Chang et al., 2009; Gallo et al., 2003; Deep et al., 2007
	Resveratrol	Antitumour	100 mg kg^−1^	Subcutaneous transplantation model of glioma cell line U87	Inhibition of angiogenesis	
*Silybum marianum* (L.) Gaertn	Silymarin	Antitumour	—	—	Inhibition of angiogenesis	Cheung et al., 2007, Lu et al., 2009; Agarwal et al., 2006
Red wine	Isoxanthohumol (XN)	Antitumour	—	—	Inhibition of angiogenesis	Costa et al., 2010
Beer	8-Butene naringin	Promoting angiogenesis	—	—	—	Costa et al., 2010
*Cinnamomum cassia* Presl	Cinnamic acid	Antitumour	—	—	Inhibition of angiogenesis	Hoskins, 1984; Thanekar et al., 2020
*Ficus carica* L	Vanillyl alcohol	Inhibition of angiogenesis	30 mg kg^−1^	Chick embryo chorioallantoic membrane model	Inhibition of angiogenesis	Jung et al., 2008 Jin et al., 2008

**FIGURE 6:**
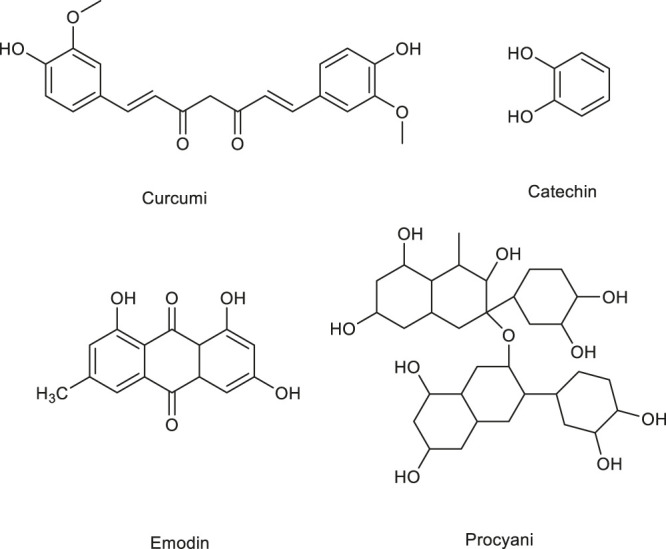
Other active compounds regulating angiogenesis in medicinal foods.

### Others Compounds

In addition to the aforementioned compounds, studies have shown that other active components extracted from food can regulate angiogenesis as well, this is consistent with the clearly defined anti-angiogenetic activity of various compounds. Therefore, although these active components have not been clearly classified yet, they can still be used in the research and development of drugs related to angiogenesis regulation (Liu et al., 2011a).

One example is the effect of black garlic on angiogenesis. In one study (Sheng et al., 2018), the authors studied the effect of black garlic on angiogenesis by establishing transgenic zebrafish *in vivo* and HUVEC *in vitro*. The transgenic zebrafish Flk-1: GFP labeled with green fluorescent protein was used as an *in vivo* evaluation model. The experimental group, control group, blank control group and vascular model group (0.2 μg ml^−1^ PTK787) were established. Positive control group (30 μg ml^−1^ Danhong injection and 0.2 μg ml^−1^ PTK787) and black garlic extract treatment group (concentration gradient of 0.2 μg ml^−1^, 0.8 μg ml^−1^, 3.2 μg ml^−1^ and 3.5 μg ml^−1^ PTK787) were cultured under normal conditions. The results showed that black garlic extract could significantly improve the pathological changes of embryo internodes and lower intestinal veins induced by PTK787, and increase the number of embryonic internodes vessels and small intestinal veins in a dose-dependent manner. When the concentration of black garlic extract increased to 3.2 μg ml^−1^, PTK787 inhibited the number of embryonic internodes vessels and inferior intestinal veins to normal. In addition, black garlic extract could enhance the activity of HUVEC and promote its growth in a dose-dependent manner. The above results showed that black garlic could promote angiogenesis by promoting HUVEC mechanism (Sheng et al., 2018).

In another study, the effect of Dalbergia odorifera extract on angiogenesis was studied (Fan et al., 2018). Using transgenic zebrafish model, the effects and mechanism of the extract of Dalbergia odorifera B3 were evaluated by observing the subintestinal angiogenesis and the damage of internode vessels in transgenic zebrafish. In this experiment, 50 g of the extract of Dalbergia odorifera B3 was extracted by reflux with 500 ml 75% ethanol, 50 g of Dalbergia odorifera decoction pieces were placed in Soxhlet extractor for 1 hour, and then the drug residue was boiled in boiling water for 1 h, concentrated to 100 ml, and then the extract was dissolved in 900 ml 95% ethanol. Next, the control group and experimental group were established. The extracts were treated with 3 μg ml^−1^, 10 μg ml^−1^ and 30 μg ml^−1^. The results showed that the extract of Dalbergia odorifera B3 had a certain promoting effect on the capillary germination of transgenic zebrafish, and the effect of 30 μg ml^−1^ was the best. These results indicate that the extract of Dalbergia odorifera B3 can promote angiogenesis and repair vascular injury induced by VEGFR kinase inhibitor II, and its mechanism is related to the promotion of VEGFR mRNA expression (Table 7).

**TABLE 7:** Other components with vascular regulation.

Source	Bioactive ingredient	Efficacy	Dose	Experimental model	Mechanism	Ref
Black garlic	Black garlic extract	Prevention and treatment of cardiovascular diseases	3.2 μg ml^−1^	Biological model experiment of zebrafish embryo	Promoting angiogenesis	Sheng et al., 2018
*Centella asiatica* (L.) Urban	Asiaticoside	Promoting skin wound healing	—	—	Enhancing the content of VEGF	Wu, 2019
*Isodon eriocalyx* (Dunn) Hara	Eriocalyxin B	Antitumour	100 μg ml^−1^	Biological model experiment of zebrafish embryo	Anti-angiogenesis	Ban et al., 2010; Li-Li et al., 2010
*Rehmannia glutinosa* (Gaert.) Libosch. ex Fisch. et Mey	Catalpol	Anti-myocardial infarction	—	—	Activate EPCs and activate Notch1 signaling pathways to promote angiogenesis	Zeng et al., 2013
*Vitis vinifera* L	3,5,7-Trihydroxylchromogenone	Antitumour	—	—	Anti-angiogenesis	Wei et al., 2014
	Nectandrin A	Antitumour	—	—	Anti-angiogenesis	Wei et al., 2014
*Panax notoginseng* (Burkill) F. H. Chen ex C. Chow & W. G. Huang	Trilinolein	Protection of the cardiovascular system	—	—	Promoting angiogenesis	Chan et al., 2002
*Hedyotis diffusa* Willd	Extract from Hedyotis diffusa	Antitumour	—	—	Down regulation of MMP-2 and MMP-9 protein expression inhibits angiogenesis	Liu et al., 2011b
*Helianthus annuus *L	Ethyl acetate part of sunflower	Antitumour	—	—	Inhibit VEGF secretion, inhibit VEGF induce endothelial cell expression	Wang et al., 2008
Black rice	Black glycosides	Antitumour	—	—	Blocking HER-2/neu and downstream EGFR/Ras/MAPK signaling pathways	Yu et al., 2010b
*Chrysanthemum coronarium* L	Campesterol	Antitumour	—	—	Inhibition of angiogenesis	Choi et al., 2007
*Perilla frutescens* (L.) Britt	Perilla leaf extract PLE	Anti-tuberculosis enteritis	100 mg g^−1^	A mouse model of pneumonia induced by dextran sulfuric acid	Inhibition of angiogenesis	Lee et al., 2020
*Kaempferia galanga* (Wall)	Ethyl *p*-methoxycinnamate	Antiangiogenic diseases	120 μg	Chick embryo chorioallantoic membrane model	Inhibition of tyrosine kinase, thereby inhibiting angiogenesis	Ekowati et al., 2015
*Aloe vera*	Trans-ethyl *p*-methoxycinnamate	Antiangiogenic diseases	—	—	Blocking bFGF induced angiogenesis	He et al., 2012b
*Opuntia dillenii* (Ker Gawl.) Haw	Cactus extract	Antitumour	—	—	Depress VEGF expression and angiogenesis	Sohail et al., 2014
*Cucurbita pepo* L	*α*-thujone	Antitumour	—	—	Depress VEGF expression and angiogenesis	Torres et al., 2016
	*β*-thujone		—	—		
*Prunella vulgaris* L	Ethanol extract from Prunella vulgaris	Antitumour	—	—	To inhibit STAT3 signaling pathway and down-regulate VEGF-A and VEGFR-2 expression	Wei et al., 2014
*Dioscorea polystachya* Turczaninow	*Ardisia crispa* roots ethanolic extract (ACRH)	Diseases associated with inflammation	100 mg kg^−1^	Balloon granuloma model in mice	Inhibition of cyclooxygenase and antiangiogenesis	Hamsin et al., 2014, Hamsin et al., 2013
	Quinone-rich fraction (QRF)		100 mg kg^−1^			
*Oncorhynchus mykiss* (Walbaum, 1792)	Chlorophyll	Antitumour	4 mg kg^−1^	Carcinogenesis model of hamster cheek pouch (HB P) induced by 7,12-dimethylbenzoanthracene	To inhibit the expression of angiogenic factor HIF-1*α*、VEGF and VEGFR2	Siddavaram et al., 2012, Chiu et al., 2005
*Salvia miltiorrhiza* Bunge	Salvianolic acid	Antiangiogenesis	1.2 mg L^−1^	Chick embryo chorioallantoic membrane model	Antiangiogenesis	Shi et al., 2013; Chimploy et al., 2009; Thiyagarajan et al., 2012; Simonich et al., 2008
*Ginkgo biloba* L	Ginkgo biloba extract	Antitumour	50 mg·(kg·d)^−1^	Lung adenocarcinoma in mice model	Reducing the expression of VEGF, improve theexpressi on of TSP-1	Zhong et al., 2016
*Dalbergia odorifera *T. Chen	Dalbergia odorifera extract	Promoting angiogenesis	30 μg ml^−1^	Biological model experiment of zebrafish embryo	Could repair the vascular damage of ISVs induced by VEGF receptor kinase inhibitor Ⅱ (VRI), and up regulate the decrease of KDR, kdrl and Flt-1 mRNA levels induced by VEGF receptor kinase inhibitor Ⅱ	Fan et al., 2018

**FIGURE 7:**
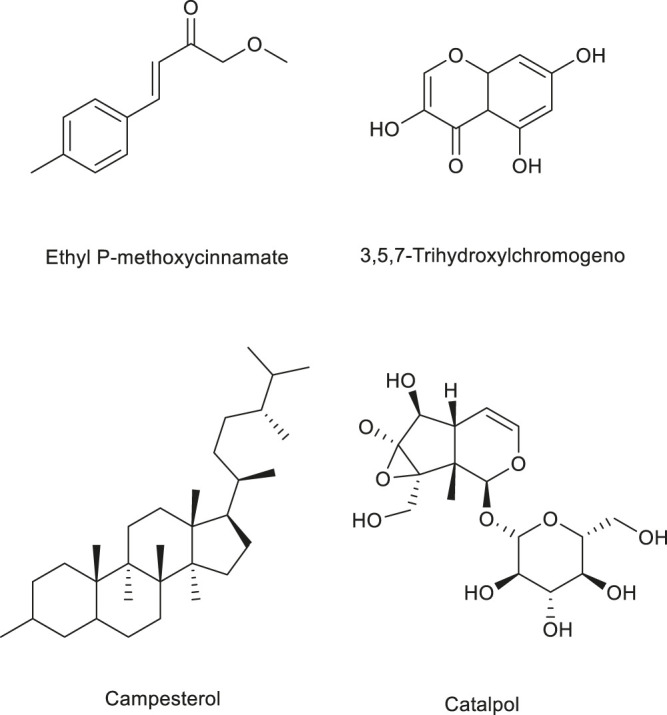


## Mechanism of Which Medicinal Food Regulates Angiogenesis

Studies have shown that angiogenesis is associated with many diseases (Qu et al., 2018a). In terms of angiogenesis regulation, the active components of medicinal foods mainly inhibit and promote angiogenesis by altering the corresponding signal transduction pathways. The VEGF and VEGFR are the two main regulatory factors the signal transduction of which is affected, thereby promoting regulatory effects. The VEGF, also known as vascular permeability factor, appears to be one of the key growth factors participating in physiological and pathological angiogenesis. This factor is present in many human tumors and may contribute to vascular hyperpermeability and enhanced angiogenesis (Dvorak et al., 1991; Kim et al., 1993; Teleanu et al., 2019). Blocking the VEGF expression by antibodies or antisense strategies has been shown to inhibit the growth of some tumors; overexpression enhances tumorigenesis. The mechanism of angiogenesis regulation by food and its active components is analysed in the following sections.

### Regulatory Mechanism of Angiogenesis Inhibition

The anti-angiogenic effect of the active components of medicinal foods is mainly achieved by inhibiting the transduction pathway of the corresponding angiogenesis signal. And mainly focuses on tumor angiogenesis and can be used to treat a small number of other angiogenesis-induced diseases.

As a study showed that tumor growth is dependent on vasculature (Folkman, 1971), the treatment of tumors has mainly focused on regulating tumor vascular growth and angiogenesis. Inhibiting the growth and migration of vascular endothelial cells, as well as regulating angiogenic factors, is one of the basic anti-angiogenic mechanisms of food active components. The specific mechanisms are directly inhibiting the proliferation and migration of vascular endothelial cells, inhibiting extracellular matrix metalloproteinase (MMP) activity, inhibiting the signal transduction of tumor angiogenic factors, and promoting the expression of tumor angiogenesis inhibitors (Lv et al., 2014).

Many studies have shown that imposing a restriction on total food intake or energy inhibits tumorigenesis (Incio et al., 2016). For example, chlorogenic acid from *Lonicera japonica* extract can inhibit the expression of MMP-9 and MMP-2 by down-regulating the COX-2-MMP signaling pathway (Cai et al., 2019); curcumin from Curcuma longa extract can inhibit tumor angiogenesis by inhibiting the proliferation of vascular endothelial cells and the expression of angiogenesis promoting factors (Gao et al., 2016b); rosmarinic acid extracted from rosemary can inhibit angiogenesis and achieve an anti-tumour effect by inhibiting VEGF expression and IL-8 release from related cells (Huang and Zheng, 2006).

### Regulatory Mechanism of Angiogenesis Promotion

In recent years, studies have shown that VEGF can promote endothelial cell division and proliferation, increase vascular permeability, and regulate thrombosis. Therefore, VEGF is closely related to the occurrence and development of certain cardiovascular diseases (Xie and Li, 2019; Qu et al., 2018b).

The active components extracted from *Kaempferia galanga*, ethyl p-methoxycinnamate and ethyl trans-p-methoxycinnamate, are used to treat angiogenic diseases by inhibiting the tyrosine kinase inhibitor of angiogenesis and blocking bFGF-induced angiogenesis (Jung et al., 2010; He et al., 2012a).

Cardiovascular diseases are mainly treated through the promotion of angiogenesis. For example, ginsenoside Rb1 promotes angiogenesis and improves heart function by promoting HIF1A activity (You et al., 2019; Kimura et al., 2006). Catalpol extracted from *Rehmannia glutinosa* can activate endothelial progenitor cells and the Notch1 signaling pathway, promote angiogenesis, and prevent myocardial infarction (Zeng et al., 2013).

The active food component-induced angiogenic regulation is not only a means to treat tumors and cardiovascular diseases, but also has therapeutic effects on other angiogenesis-related diseases, as it promotes the healing of skin wounds and eye diseases caused by blood vessels.

Beta-sitosterol, an active component of aloe vera, has been found to promote wound healing by promoting angiogenesis (Moon et al., 1999). Asiaticoside promotes skin wound healing by increasing the VEGF content (Wu, 2014). Fucoidan can not only inhibit the mitosis and chemotaxis of VEGF165 in HUVEC by blocking the binding of VEGF to its cell surface receptors but also has an anti-coagulation effect by inhibiting the expression of VEGF (Satoru et al., 2003; Xin et al., 2017).

## Conclusions and Future Perspectives

The aforementioned studies indicate that a medicinal diet often has a good regulatory effect on angiogenesis. They can regulate by inhibiting and promoting the transmission of different angiogenesis-related signals and can be used in the treatment of angiogenic diseases such as cardiovascular diseases and tumors. Cardiovascular and cerebrovascular diseases and tumors are two conditions that have attracted much attention since the 20th century. In the past, studies on these mainly focused on vasodilators and antineoplastic drugs. However, clinical trials have shown that both drug types have clinical limitations, including drug resistance, unclear treatment window, and lack of effective biomarkers. In addition, long-term human use of these drugs can lead to the development of drug resistance and dependence (Fu et al., 2020). Compared with traditional medicine, medicinal food is safer and more easily accepted by people, and the natural chemical products present in it are safer and more reliable. Therefore, since the first discovery that medicinal food and its active ingredients have a good regulatory effect on angiogenesis, the role of food in regulating angiogenesis in disease treatment has attracted extensive attention (He, 2019).

With the advancement of research, the angiogenic regulatory effect of active ingredients in medicinal foods has been gradually revealed. Therefore, it is incredibly important to understand the pathogenesis and regulatory mechanism of angiogenesis to use active ingredients in foods for mediating their angiogenic effect. This will also contribute to a solid foundation for the research and development of therapeutic drugs for vascular diseases based on the characteristics of medicinal foods and their active ingredients.

Some experimental studies have shown that medicinal foods and their active ingredients can regulate angiogenesis and be directly used to treat angiogenic diseases. However, they also have broad application prospects in the field of research, development, and synthesis of angiogenesis-regulating drugs or health products. Although it is safer to treat vascular diseases with medicinal foods and their active ingredients than with traditional medicines, the currently available knowledge has many limitations. First, the current experimental research is only limited to the laboratory construction of animal experimental models or basic pharmacological activity experiments, and there is no regulation on the follow-up clinical trials and other studies. Second, the research on active ingredients of medicinal food is obviously insufficient. Many studies have shown that active ingredients in foods can regulate angiogenesis, but no studies have specified a dose. Most studies only indicate a certain dose range, and the measurement is not uniform, even though the correct dosage is extremely important. Third, the regulatory mechanism of medicinal foods and their active ingredients on angiogenesis is not clear as such foods constitute many ingredients. As previous studies have shown that VEGF is highly concentrated in tumor cells, the inhibition of VEGF-related receptors may be an effective treatment for such diseases (Ge et al., 2019). However, owing to the many regulatory factors, it is not simple to determine the main mechanism of action of medicinal foods and their active ingredients. Finally, an important research problem that needs to be solved is the lack of quality control in these studies owing to the influence of many factors on the active ingredients of medicinal foods. Therefore, the follow-up study of angiogenesis-related factors and their receptors is crucial, as it will provide more reliable targets for the future research and development of new drugs. Moreover, it is particularly important for later clinical trials, which can provide more powerful evidence for later drug development.
